# Main Targets of Interest for the Development of a Prophylactic or Therapeutic Epstein-Barr Virus Vaccine

**DOI:** 10.3389/fmicb.2021.701611

**Published:** 2021-06-22

**Authors:** Vincent Jean-Pierre, Julien Lupo, Marlyse Buisson, Patrice Morand, Raphaële Germi

**Affiliations:** ^1^Laboratoire de Virologie, Institut de Biologie et de Pathologie, CHU de Grenoble Alpes, Grenoble, France; ^2^Institut de Biologie Structurale, UMR 5075, CEA, CNRS, Université Grenoble Alpes, Grenoble, France

**Keywords:** Epstein-Barr virus (human gammaherpesvirus 4), EBV-associated cancer, glycoproteins, infectious mononucleosis, latency, lytic cycle, prophylactic vaccine, therapeutic vaccine

## Abstract

Epstein-Barr virus (EBV) is one of the most widespread viruses in the world; more than 90% of the planet’s adult population is infected. Symptomatic primary infection by this *Herpesviridae* corresponds to infectious mononucleosis (IM), which is generally a benign disease. While virus persistence is often asymptomatic, it is responsible for 1.5% of cancers worldwide, mainly B cell lymphomas and carcinomas. EBV may also be associated with autoimmune and/or inflammatory diseases. However, no effective treatment or anti-EBV vaccine is currently available. Knowledge of the proteins and mechanisms involved in the different steps of the viral cycle is essential to the development of effective vaccines. The present review describes the main actors in the entry of the virus into B cells and epithelial cells, which are targets of interest in the development of prophylactic vaccines aimed at preventing viral infection. This review also summarizes the first vaccinal approaches tested in humans, all of which are based on the gp350/220 glycoprotein; while they have reduced the risk of IM, they have yet to prevent EBV infection. The main proteins involved in the EBV latency cycle and some of the proteins involved in the lytic cycle have essential roles in the oncogenesis of EBV. For that reason, these proteins are of interest for the development of therapeutic vaccines of which the objective is the stimulation of T cell immunity against EBV-associated cancers. New strategies aimed at broadening the antigenic spectrum, are currently being studied and will contribute to the targeting of the essential steps of the viral cycle, the objective being to prevent or treat the diseases associated with EBV.

## Highlights

- EBV is an oncogenic virus with tropisms for B cells and epithelial cells.
- gp350 vaccines reduce the risk of IM without preventing EBV infection.
- gp350/220, gH, gL, gp42, and gB glycoproteins are targets for neutralizing antibodies.
- Latency proteins are involved in EBV oncogenesis.
- Some lytic cycle proteins may contribute to EBV oncogenesis.
- New vaccine candidates combine latency and lytic cycle antigens.

## Introduction

Epstein-Barr virus (EBV), also known as Human gammaherpesvirus 4, belongs to the *Herpesviridae* family and the *Gammaherpesvirinae* sub-family. This enveloped double-stranded DNA virus with a diameter of 150–200 nm, was discovered in 1964; it initially arose from Burkitt lymphoma tumor cells (Epstein et al., 1964; Mui et al., 2019).

Interhuman transmission of the virus is essentially salivary. In developing countries, almost all children are infected before four; in developed countries, less than 50% of children between five and ten are EBV-seropositive (Niederman and Evans, 1997). While primary EBV infection is more often asymptomatic, it can in some cases be associated with infectious mononucleosis (IM), which is characterized by angina, lymphadenopathy, fever, and fatigue. The later the occurrence of primary infection in the life of an individual (during adolescence or adulthood), the greater the risk of developing IM (Cohen, 2015). Though it is generally a benign disease, in 1% of cases it can entail complications that may be serious (encephalitis, myocarditis, hepatic complications…) or disabling; in 10% of patients, chronic fatigue lasts up until 6 months after viral infection (Dasari et al., 2017).

Subsequent to primary infection, EBV persists for a lifetime in the memory B lymphocytes of the infected host, albeit generally without pathological consequences on the individual. However, viral persistence can be associated with the development of cancer. More precisely, EBV is classified in group 1 of human carcinogens. It is the first human oncogenic virus to have been discovered and to this day, it remains the only human pathogen that can immortalize and transform cells *in vitro* (Niedobitek, 1999). Given its B cell tropism, EBV infection can be associated not only with B cell lymphomas (Hodgkin, Burkitt lymphoma), but also with the lymphoproliferative disorders observed in a context of immunodepression, particularly in solid organ transplant patients or in hematopoietic stem cell recipients during the first year following transplantation (Shannon-Lowe et al., 2017). Moreover, given its epithelial cell tropism, EBV is the source of nasopharyngeal cancer and can be associated with gastric carcinomas (Dasari et al., 2019). However, as the virus does not persist in epithelial cells, epithelial tumors arise from the latent viral reservoir present in B cells (Brooks et al., 2016). An estimated 200,000 new cases of EBV-induced cancers occur every year, representing 1.5% of the cancers reported worldwide (Holmes, 2014), and they are responsible for approximately 164,000 deaths a year (Khan et al., 2020).

Epstein-Barr virus may also be associated with the development of inflammatory and autoimmune diseases such as multiple sclerosis (Levin et al., 2010), systemic lupus erythematosus (Ascherio and Munger, 2015), rheumatoid arthritis (Balandraud and Roudier, 2018), and the Sjögren syndrome (Sorgato et al., 2020), but these associations remain controversial.

As it infects over 90% of the world population, EBV is one of the most widespread viruses throughout the planet; however, to this day, no effective treatment or anti-EBV vaccine are available (Pei et al., 2020).

A prophylactic vaccine, which would prevent EBV infection and thereby provide protection against associated diseases, or a therapeutic vaccine, which would directly treat the pathologies associated with EBV, would undeniably be of considerable interest for public health.

The oncogenic potential of EBV precludes its being used in vaccinal projects in an attenuated or inactivated form. That is one reason why development of an anti-EBV vaccine presupposes optimal knowledge of the different elements contributing to the virus’s life cycle, namely the viral and cellular proteins implicated in the entry of the virus into host cells (prophylactic vaccines), and the proteins involved in viral persistence (therapeutic vaccines).

## Virus Entry and Prophylactic Vaccines

During primary infection, the virus crosses the mucosal epithelial cell barrier by transcytosis and then infects the B cells in the submucosal secondary lymphoid tissues (Tangye et al., 2017).

Epstein-Barr virus entry proceeds in two successive steps, the first of which consists in the tethering of the virus to target cells; viral glycoproteins and cell-adhesion receptors bring nearer and concentrate the virus at the target cell surfaces without triggering cell fusion mechanisms. Contrarily to most of the other enveloped viruses, which utilize only one or two glycoproteins, several viral envelope glycoproteins play a key role in the mechanism of EBV entry into B cells and epithelial cells. The second step consists in the fusion of the virus with the endocytic membrane of the B cells or the plasma membrane of the epithelial cells, a process involving proteins of the fusion machinery highly conserved within the *Herpesviridae* family and cellular entry receptors (Connolly et al., 2011).

Interestingly, the mechanisms of virus entry differ between B cells and epithelial cells. While the entry of EBV into B cells is carried out by endocytosis, EBV enters the epithelial cells by direct fusion of the viral and cellular membranes (Miller and Hutt-Fletcher, 1992).

### The Entry of EBV Into B Cells

Glycoprotein 350/220 (gp350/220), expressed on the EBV envelope, is presented in two forms, one in 350 kDa and the other in approximately 220 kDa (alternative splicing of the same primary RNA transcript). Gp350/220 is strongly implicated in the infection of B cells while its involvement is limited in the infection of epithelial cells.

**FIGURE 1:**
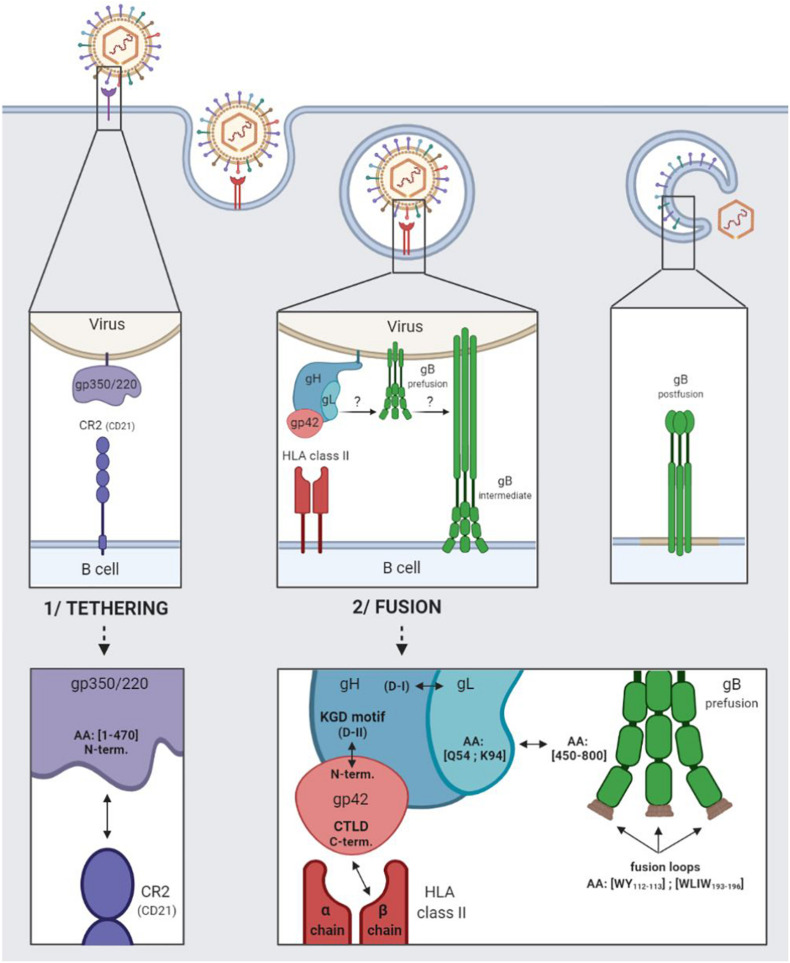
EBV entry into B cells by endocytosis. 1/Tethering of the virus to the B cell involving the viral gp350/220 glycoprotein and the cellular CR2 receptor. 2/Fusion of the viral and cellular membranes involving the viral gH/gL, gp42, and gB glycoproteins and the cellular HLA class II.

The entry of EBV into B cells begins with the tethering of gp350/220 via its N-terminal residues 1–470 to the complement receptor type 2 (CR2), also termed CD21, or to the complement receptor type 1 (CD35 or CR21) (Connolly et al., 2011). Figure 1 outlines in detail the relevant interactions.

Following which, glycoproteins H (gH), L (gL), and 42 (gp42) come into play, regulating the activation of glycoprotein gB, which plays a direct role in membrane fusion (Heldwein, 2016). Taken together, these four glycoproteins are involved in the fusogenic mechanism enabling EBV to enter into B cells.

The gH glycoprotein consists in four major domains ranging from D-I (N-terminal) to D-IV (C-terminal). The gL glycoprotein interacts with the D-I domain to form the heterodimeric gH/gL complex (also termed gp85/gp25) (Matsuura et al., 2010). The gp42 N-terminal part is wrapped around the D-II, D-III, and D-IV domains of gH, and tethered to the KGD-binding motif (present in the D-II domain) (Möhl et al., 2019).

Following the tethering of gp350/220 to CR2, gp42 C-type lectin domain (CTLD situated in the C-terminal) interacts specifically with the β chains of the human leukocyte antigen (HLA) class II molecules (Kirschner et al., 2009; Sathiyamoorthy et al., 2014). The interaction enlarges the hydrophobic pocket of the gp42 CTLD, thereby enabling the gH/gL complex [*via* the glutamine (Q) 54 and lysine (K) 94 residues of the glycoprotein gL] to be tethered to the pre-fusion form of gB (gB residues 450–800) (Mohl et al., 2016). The binding of gH/gL to gB triggers the conformational change of gB into an intermediate form, enabling the insertion of gB fusion loops (hydrophobic residues WY_112__–__113_ and WLIW_193__–__196_) into the B cell membrane. Fusion of the viral and cellular bilayers leads to the nucleocapsid release into the cytoplasm of the target cells, as a result of which, gB takes on a stable, post-fusion conformation (Möhl et al., 2017). As of now, only the post-fusion gB structure is known.

### The Entry of EBV Into Epithelial Cells

The CR2, to which gp350/220 binds for entry into B cells, is not constantly expressed in epithelial cells. This makes the role of gp350/220 minor in EBV entry into these epithelial cells.

However, the binding of the virus to CR2-negative epithelial cells is five times weaker than its binding to CR2-positive epithelial cells (Chen and Longnecker, 2019).

**FIGURE 2:**
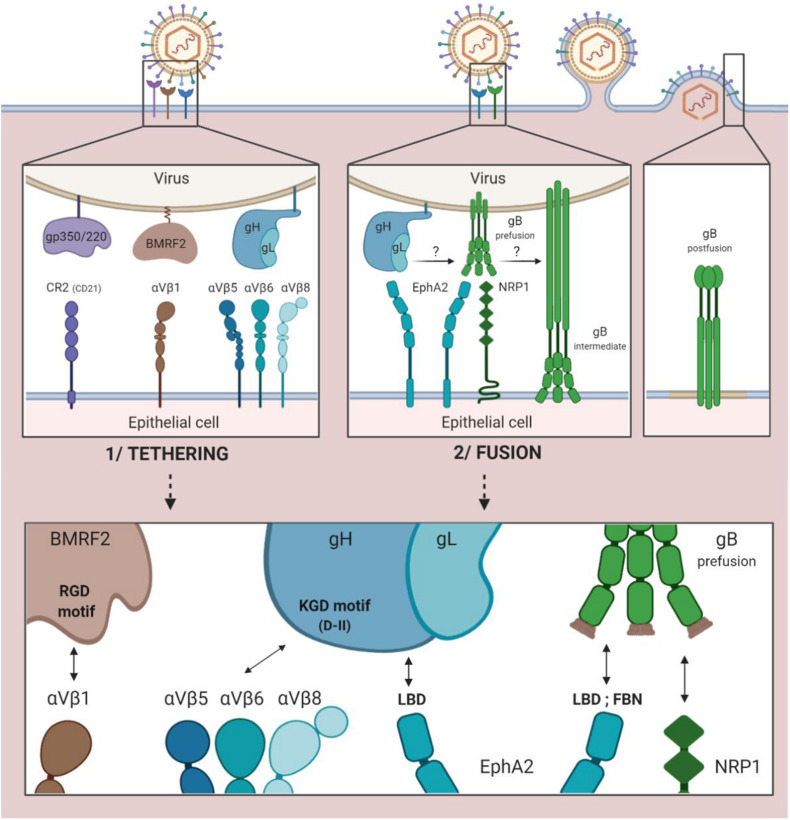
EBV entry into epithelial cells by direct fusion. 1/Tethering of the virus to the epithelial cell involving the viral gp350/220, BMRF2 and gH/gL glycoproteins and the cellular CR2 receptor (case of CR2+ epithelial cells) and cellular integrins (αVβ1, 5, 6, and 8). 2/Fusion of viral and cellular membranes involving the viral gH/gL and gB glycoproteins and the cellular EphA2 and NRP1 proteins.

While interaction between the cellular integrin αVβ1 and the RGD integrin binding motif (arginine/glycine/aspartic acid) of the viral protein BMRF2 is a first step toward facilitated entry, it does not necessarily take place (Figure 2; Connolly et al., 2011). The BMRF2 protein can also form a complex with the viral protein BDLF2 (not represented in Figure 2). BDLF2 is a type II membrane protein that participates in the rearrangement of the cellular actin network, thereby increasing contacts between the different cells and favoring circulation of the virus from one cell to another (Gore and Hutt-Fletcher, 2009).

Due to the KGD motif of gH, the gH/gL complex of the virus is subsequently tethered to the cellular integrins αVβ5, αVβ6, and αVβ8 (Chesnokova and Hutt-Fletcher, 2011; Connolly et al., 2011). Indeed, the KGD motif is capable of being bound competitively to either the epithelial cellular integrins or to gp42, and the double functionality of the KGD motif of gH is what renders gp42 a major actor in the cellular tropism of EBV (Chen et al., 2014). More precisely, the presence of gp42 masks the KGD motif of gH to epithelial cell integrins and inhibits the fusion of the virus with these cells.

The following step involves the cellular protein EphA2 (ephrin receptor tyrosine kinase A2). The ectodomain of this protein consists in four regions: an LBD region (ligand binding domain) that can be bound to gH/gL and gB, a region rich in cysteine and two fibronectin regions (FBN) that interact with gB. This step is essential, given the fact that in the absence of EphA2, fusion between viral and cellular membranes is reduced by more than 90% and infection by 85% (Chen et al., 2018). Conversely, its overexpression facilitates fusion and infection of the epithelial cells (Chen and Longnecker, 2019).

The fusion process previously described with regard to B cells can consequently be initiated due to interaction between gH/gL and the pre-fusion form of gB, thereby enabling the entry of the virus into epithelial cells.

Another cellular actor has been characterized as having a role in the fusion of EBV with the epithelial cells. Neuropilin 1 (NRP1) is a cellular protease of which the overexpression significantly increases the infection of epithelial cells by EBV; when inhibited, on the other hand, it reduces infection by 50% (Wang et al., 2015). Of note, NRP1 is very weakly expressed by B cells as compared to epithelial cells.

### The Tropism of EBV Infection

While gp42 does not play a part in the mechanism of entry of EBV into the epithelial cells, it nevertheless mediates the cellular tropism of the virus (Möhl et al., 2019). More precisely, when neosynthesized virions are exported from B cells, gp42 remains sequestered due to its interaction with HLA class II molecules. As a result, the mature virions exported from B cells are, for the most part, deficient in gp42, and cannot reinfect another B cell (Mohl et al., 2016). Conversely, the absence of gp42 at the surface of the virions promotes infection of the epithelial cells. When the neosynthesized virions are exported from the epithelial cells, which do not contain HLA class II molecules, gp42 is not sequestered and the mature virions are enriched in gp42, a factor facilitating their entrance into the B cells, and so on and so forth (Sathiyamoorthy et al., 2016).

### Other EBV Surface Glycoproteins

**FIGURE 3:**
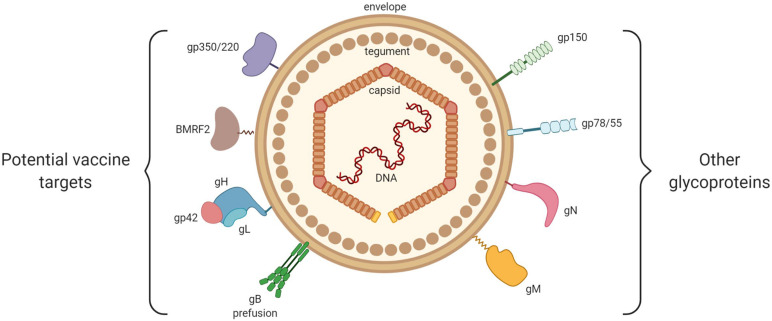
General structure of EBV. From inside to outside are represented: the EBV double-stranded DNA, the icosahedral capsid, the tegument and the envelope. On the left are shown the viral envelope glycoproteins that play a key role in EBV entry into host cells (gp350/220, BMRF2, gH/gL, gp42, and gB). On the right are shown the viral glycoproteins with little or no involvement in EBV entry (gp150, gp78/55, gN, and gM).

Other than the just-mentioned glycoproteins, which actively participate in the entrance of the virus into host cells, there exist other glycoproteins that are probably less relevant as possible targets in a vaccine project, and of which the exact roles in the viral cycle remain to be clarified (Johannsen et al., 2004; Figure 3).

Among them, we may cite glycoprotein N (gN), which is encoded by the BLRF1 gene, and glycoprotein M (gM), which is composed of several transmembrane domains and is encoded by the BBRF3 gene. Highly conserved in the *Herpesviridae* family, these glycoproteins form the gN/gM complex (Lake and Hutt-Fletcher, 2000). Non-expression of the gN/gM complex reduces the binding of the virus to target cells, blocks dissociation of the viral capsid from the cellular membranes (thereby preventing its migration toward the nucleus), and limits the release of new virions (Lake and Hutt-Fletcher, 2000).

Encoded by the BDLF3 gene, glycoprotein 150 (gp150) is capable of binding to the heparan sulfate present at the surface of the epithelial cells, binding that does not provoke the infection of target cells (Chesnokova et al., 2016).

Encoded by the BILF gene, glycoprotein 78/55 (gp78/55) does not seem to have a role in virus entrance (Mackett et al., 1990).

### The Main Anti-gp350/220 Prophylactic Vaccines

**FIGURE 4:**
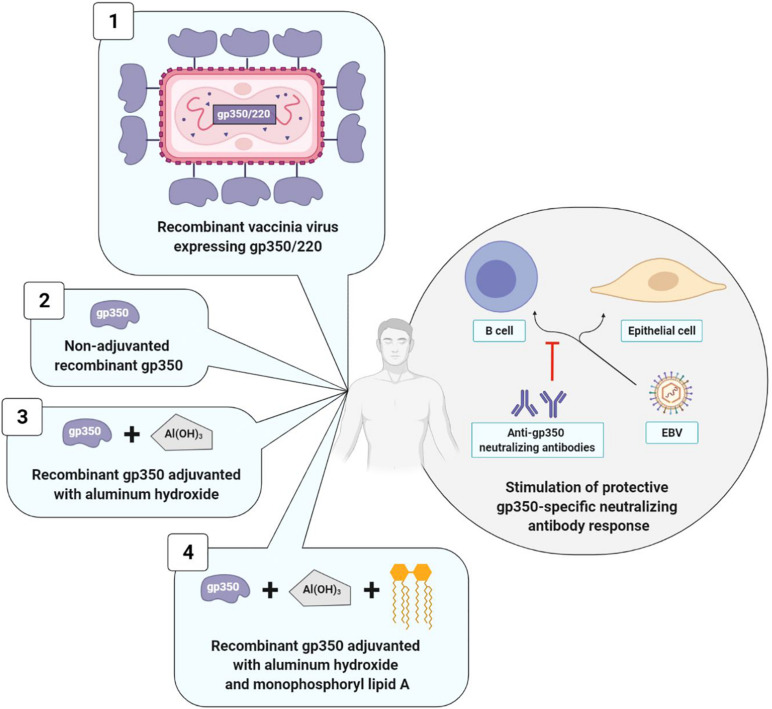
Prophylactic vaccine strategies using gp350/220 as antigen. The four types of strategies elicit a neutralizing anti-gp350 immune response. Strategies with adjuvant (3 and 4) induce higher anti-gp350 antibody titers than strategy without adjuvant (2). Strategy with monophosphoryl lipid A (4) induce a higher and long-lasting neutralizing anti-gp350 humoral response than strategy without monophosphoryl lipid A (3). Data collected for strategy 1 are not comparable to those of other strategies. 1, Gu et al., 1995; 2, Moutschen et al., 2007; 3, Moutschen et al., 2007; Rees et al., 2009); 4, Moutschen et al., 2007; Sokal et al., 2007.

The initial and principal approaches to prophylactic vaccination naturally targeted gp350/220, which is the most abundant glycoprotein on the surface of EBV and infected cells and represents the main target of anti-EBV neutralizing antibodies (Cohen et al., 2011). The different vaccinal approaches are summarized in Figure 4.

The first anti-EBV vaccine clinical trial was developed in 1995 by a Chinese team that used as its vector, a live vaccinia virus (VV) expressing gp350/220. The vaccine was administered in a single dose to: (i) EBV and VV-seropositive adults; (ii) EBV-seropositive juveniles who were nonetheless VV-seronegative; (iii) children who were seronegative for the two viruses. The outcomes were analyzed 16 months after vaccine administration. In adults, due to their anti-VV immunity, the VV did not multiply and the vaccine had no effect. On the contrary, in vaccinated juveniles, anti-EBV neutralizing antibody titers increased, and among the nine seronegative vaccinated children, only three were EBV-infected, whereas all 10 of the non-vaccinated children were (Gu et al., 1995). Due to the biosafety standards imposed in vaccinology, this initial clinical study was not pursued.

It was only in 2007 that Moutschen et al. (2007) published the results of two new clinical trials (one phase I, and the other phase I/II), which were carried out with 148 healthy adults. Both of these studies demonstrated the safety and immunogenicity of a soluble gp350 recombinant monomer, associated or not with an adjuvant. This candidate vaccine was obtained from CHO (Chinese Hamster Ovary) cells expressing the gp350/220 gene having undergone splice site mutation, thereby preventing formation of the gp220 isoform. The preparations associating an adjuvant (aluminum hydroxide alone or combined with monophosphoryl lipid A) induced anti-gp350 antibody titers significantly higher than preparations without an adjuvant and the three tested candidate vaccines were well-tolerated (Moutschen et al., 2007).

In order to confirm the safety and immunogenicity of this anti-EBV vaccine and to assess its efficacy, Sokal et al. (2007) carried out a phase II, double blind, randomized trial. The soluble gp350 recombinant monomer associated with aluminum hydroxide/monophosphoryl lipid A was distributed in three doses to 181 EBV-seronegative young adults and compared to a placebo consisting in aluminum hydroxide alone. A durably neutralizing anti-gp350 antibody response was observed over a period exceeding 18 months in 98.7% of the vaccinated patients. While this humoral response did not suffice to prevent EBV infection, it significantly reduced (78%) IM incidence in the vaccinated group; IM occurrence was 4.8 times higher in the placebo group than in the vaccinated group (Sokal et al., 2007). In conclusion, the anti-gp350 neutralizing antibodies seemed to attenuate the severity of the disease associated with EBV infection; on the other hand, they did not prevent the infection itself (Tangye et al., 2017).

Two years later, another phase I study assessed the effect of the gp350 recombinant monomer associated with aluminum hydroxide in the prevention of post-transplant lymphoproliferative disease (PTLD) in 16 EBV-seronegative children with chronic renal disease who were awaiting transplantation. The objective of this vaccine was to stimulate anti-EBV humoral immunity prior to transplantation in view of reducing PTLD appearance and severity. The two tested doses of the vaccine (12.5 and 25 μg) were well-tolerated and induced similar anti-gp350 antibody titers in all of the vaccinated patients; however, only four patients developed anti-gp350 neutralizing antibodies. What is more, anti-gp350 immune responses (neutralizing or not) rapidly declined a few weeks after the last injection, and 26 weeks after transplantation, the EBV viral loads measured in the blood by PCR were similar between vaccinated and non-vaccinated children. A preventive effect of the vaccine on PTLD consequently appears unlikely (Rees et al., 2009).

## Viral Latency, Viral Replication, and Therapeutic Vaccines

The second anti-EBV vaccine strategy consists in stimulating T cell immunity to EBV and/or restoring immune control in patients with EBV-associated cancers. So-called therapeutic vaccines rely primarily on the latency proteins implicated in the transformation and immortalization of infected cells and, consequently, the development of cancers. Some proteins in the lytic cycle also play a major role in the establishment of viral persistence. Therapeutic vaccines could represent an alternative to the radiotherapies and chemotherapies usually applied in treatment of EBV-associated cancers, which are responsible for considerable adverse effects.

Development of therapeutic vaccines necessitates knowledge of several different steps and of the proteins involved in the latency cycle and the EBV replication cycle in infected individuals.

### The EBV Latency Cycle

**FIGURE 5:**
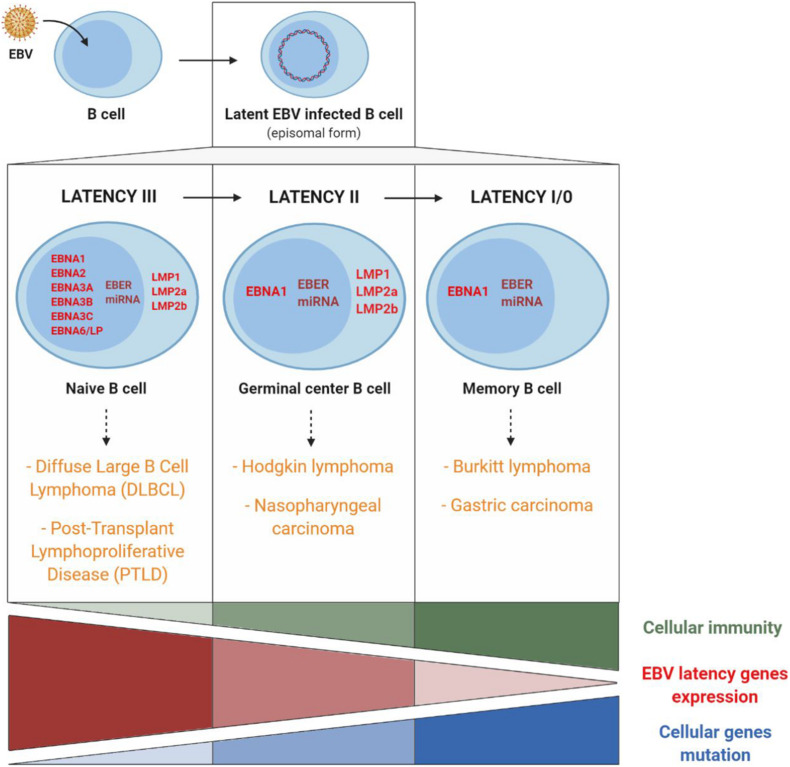
EBV latency gene expression profiles and involvement of cellular immunity, of EBV latency gene expression, and of cellular gene mutations in EBV-associated cancers. Cancers associated with EBV type III latency are mainly found in immunocompromised patients. Conversely, cancers associated with EBV type I latency are mainly found in immunocompetent patients and are related to host cell gene mutations. Adapted from Rickinson and Kieff (2013) and Lupo et al. (2019).

After primary infection, EBV persists as an episomal latent form in the infected memory B lymphocytes. At this stage, there exists a balance between latency, replication of the virus in the organism and elimination of the infected cells by immune responses. Latency is characterized by the expression of viral latency genes, six of which code for nuclear proteins EBNA (Epstein-Barr nuclear antigen), three for membrane proteins LMP (latent membrane protein) and two for non-coding RNAs EBER (Epstein-Barr encoded small RNA). A large number of viral-derived micro-RNAs (miRNA) are likewise expressed (44 stemming from the BART gene and three from the BHRF1 gene) (Iizasa et al., 2020; Figure 5). These different latency proteins and non-coding RNAs of EBV contribute to the capacity of the virus to immortalize and ensure B cell proliferation *ad infinitum*. There exist different expression profiles for latency genes, enabling them to persist in B lymphocytes during cell division. These different profiles are expressed during the different natural phases of latency establishment but also in different EBV-associated cancer pathologies. In the three types of latency, EBNA1 expression allows the long-term persistence of the EBV genome as an episome and its replication by cellular DNA polymerase. During the latency phase, the virus is replicated synchronously with the infected memory B cells, which thereby become the main site of EBV persistence (Andrei et al., 2019).

Type III latency corresponds to the expression in naive B cells of all the different latency genes. This type of latency is found mainly in non-Hodgkin lymphomas such as PTLD or in EBV-associated diffuse large B cell lymphomas. In most cases, this type of lymphoma appears in immunocompromised persons in whom reduced immunosurveillance entails uncontrolled B cell proliferation, which fosters the appearance of genetic alterations and the development of cancer (Cohen, 2015).

Type II latency corresponds to the expression, in germinal centers, of EBNA1, LMP1, LMP2, non-coding EBER RNAs and miRNA and is found in EBV-associated Hodgkin lymphomas and nasopharyngeal carcinomas (NPC).

Latency I/0 is present in memory B cells, in which only EBNA1 and non-coding EBER RNAs and miRNA are expressed and is found in Burkitt lymphomas and EBV-associated gastric carcinomas.

In Hodgkin and Burkitt lymphomas and NPC, EBV expression is consequently limited to a few latency genes, and reduced host immune response is of limited importance. On the other hand, the role of cellular gene mutations is essential in the pathogenesis and the development of these EBV-associated cancers (Figure 5).

As a result, the EBNA1 protein (expressed in all the EBV-associated malignant tumors), the signaling membrane proteins LMP1 (oncogene implicated in cell transformation and survival) and LMP2 (regulator of virus reactivation from latency) as well as the non-coding RNAs (implicated in immune escape rather than cell transformation) represent promising targets for the composition of an anti-EBV therapeutic vaccine (Dasari et al., 2019; Münz, 2019).

### The EBV Lytic Cycle

As the study of EBV-associated cancers has for many years been exclusively focused on latency proteins, the role of the lytic cycle seems to have been underestimated.

The EBV lytic cycle (also known as a replicative or multiplication cycle) is divided into three sequential phases (immediate early, early and late) during which more than 80 proteins are expressed.

**FIGURE 6:**
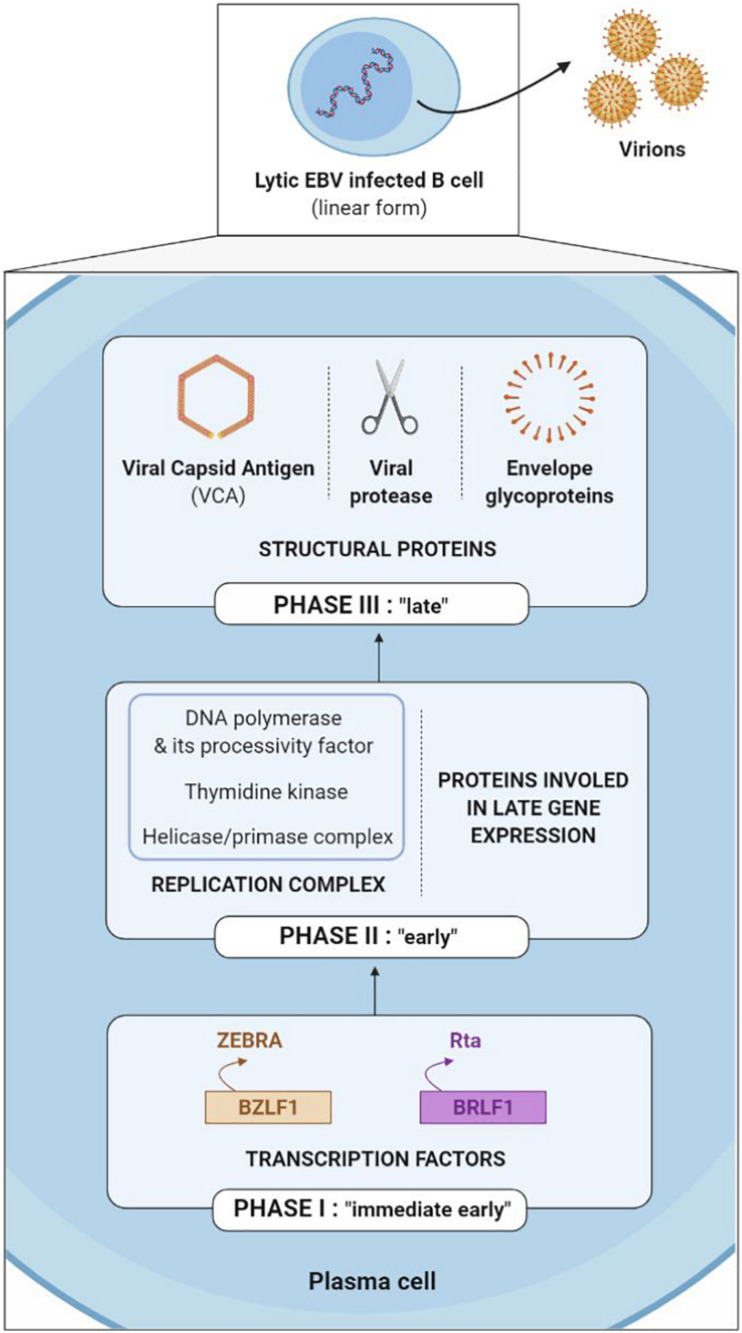
Sequential expression of genes involved in the EBV lytic cycle.

The first genes to be transcribed during the immediate early phase are the BZLF1 and BRLF1 genes, which code the ZEBRA (or Zta or Z) and the Rta (or R) transcription factors respectively. The ZEBRA and Rta proteins then activate the early gene promoters, which code for either the replication complex (including viral DNA polymerase, its processivity factor, thymidine kinase and the helicase-primase complex) or the proteins involved in late gene expression. After which, late genes are expressed, coding for structural proteins such as viral capsid antigen (VCA), the viral protease necessary for maturation of the capsid and the envelope glycoproteins; taken together, they enable construction of new viral particles (Kenney and Mertz, 2014; Figure 6).

The immediate early ZEBRA and Rta proteins are more often the target of TCD8+ cells, while the early and late proteins are more often the target of TCD4+ cells (Cohen, 2018). Thus, EBV surface glycoproteins (late proteins) are not simply the target of humoral immunity with neutralizing antibodies; they are also targeted by glycoprotein-specific CD4+ T cells, which are capable of recognizing newly EBV-infected cells (Brooks et al., 2016).

At present, numerous arguments emphasize the role of proteins expressed during the lytic cycle in the initiation and development of EBV-associated cancers. The partial success of anti-EBV immunotherapy targeting the antigens of the lytic cycle in EBV-associated cancers is one of these arguments (Münz, 2020). In addition, it has been shown that humanized mouse models developed fewer B cell lymphomas when they were infected with a deleted EBV mutant of the lytic gene BZLF1 than with a non-muted EBV (Ma et al., 2011).

As a result, lytic cycle proteins – and, more precisely, immediate early proteins – may be of interest in possible association of latency proteins with the conception of a therapeutic vaccine. A vaccine composed of the main lytic proteins could stimulate the TCD4+ and TCD8+ cellular immunity specific to these antigens and eliminate infected cells, thereby limiting the formation of new virions, as well as the infection and transformation of new cells.

### The Main Therapeutic Anti-EBV Vaccines Tested in Humans

Most of the therapeutic anti-EBV vaccines have been developed for the treatment of NPC, which represents the third most frequent cause of cancer in southern China (Straathof et al., 2005). NPC is an epithelial tumor closely associated with EBV (100% of cases) in which the tumor cells express a type II latency profile. The EBNA1 and LMP2 proteins significantly contribute to the transformation of normal cells into cancer cells. The EBNA1 protein includes epitopes that are targeted mainly by TCD4+ cells, and to a lesser extent by TCD8+ cells. Conversely, the LMP2 protein contains epitopes that are targeted mainly by TCD8+ cells, and only to a small extent by TCD4+ cells (Dasari et al., 2019).

That is why EBNA1 and LMP2 are the two mainly targeted antigens in therapeutic vaccines.

**FIGURE 7:**
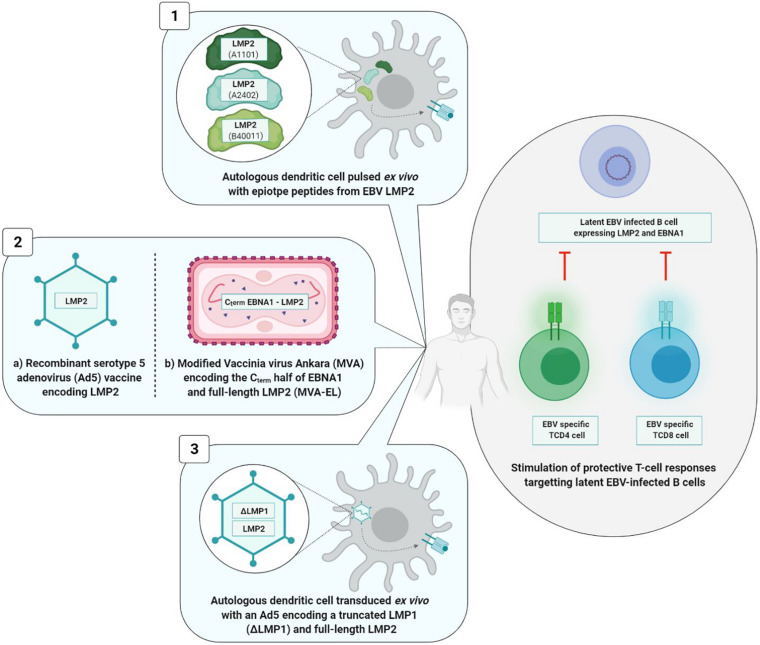
Therapeutic vaccine strategies. The three types of strategies induce a TCD8+ and/or TCD4+ cellular immune response. Viral vectors based strategies (2a and 2b) induce a higher and long-lasting cellular immunity than the strategy based on dendritic cells (1) and the mixed strategy (3). 1, Lin et al., 2002; 2a, Borovjagin et al., 2014; 2b, Hui et al., 2013; Taylor et al., 2014; 3, Chia et al., 2012.

The three therapeutic vaccine strategies are summarized in Figure 7. The first one is based on dendritic cells (DC) and the second on viral vectors, while the third is mixed.

Dendritic cells based strategy is situated at the interface of vaccination and immunotherapy. It was tested during a phase I clinical study involving 16 patients at an advanced stage of NPC. Autologous DC were initially harvested and then pulsed *ex vivo* with different LMP2 epitope peptides (A1101, A2402, or B40011) before being reinjected into the inguinal lymph nodes of patients. DC are antigen presenting cells that are of paramount importance in the induction of T cell immune response. In this Chinese study, TCD8+ immune responses directed against LMP2 epitopes were observed in nine vaccinated patients, and partial tumor reduction was noted in two of them. However, 6 months after the first injection, the LMP2-specifc TCD8+ cells had reverted to their level prior to vaccination (Lin et al., 2002).

The second vaccine technology uses a viral vector [adenovirus or modified Vaccinia virus Ankara (MVA)]. The genome of these viral vectors is modified by insertion of a DNA sequence of one or more EBV antigens. A phase I clinical study carried out in 24 advanced stage NPC patients demonstrated the safety and immunogenicity of a recombinant type 5 adenovirus (Ad5) (one of the least pathogenic for humans) expressing LMP2 protein (Borovjagin et al., 2014). Surprisingly, the vaccine significantly increased not the level of TCD8+ lymphocytes, but rather the level of TCD4+ lymphocytes alone, and only in patients vaccinated with the highest dose of the tested vaccine (Si et al., 2016).

In their respective approaches to viral vector, two teams have succeeded in improving TCD8+ immune response (Lin et al., 2002) by associating antigen EBNA1 epitopes with LMP2 epitopes and by utilizing the attenuated and recombinant MVA virus as viral vector. In fact, TCD4+ cells assume a crucial role in maintenance of a durably effective memory CD8+ T cell response (Taylor et al., 2004). Known as MVA-EL, the modified virus coded for a fusion protein containing the C-terminal motif of protein EBNA1 as well as the whole LMP2 protein.

An initial phase I clinical trial was carried out in Hong Kong in 2013 including 18 NPC patients who had been in remission for more than 12 weeks, the objective being to assess the safety and immunogenicity of the MVA-EL vaccine (Hui et al., 2013). The same clinical trial took place in the United Kingdom in 2014, involving 14 patients (Taylor et al., 2014). In 15 out of the 18 Hong Kong patients and eight out of the 14 British patients having received three intradermic MVA-EL vaccines 3 weeks apart, the T cell immune response directed against at least one of the two vaccine antigens (EBNA1 or LMP2) was increased.

Conducted in parallel, the two studies showed that the MVA-EL candidate vaccine was well-tolerated and that it increased specific TCD8+ and TCD4+ immune responses.

The third strategy is mixed; while it brings into play the DC, the tumor antigens are incorporated in an adenoviral vector.

A phase II clinical trial carried out in 19 advanced stage NPC patients assessed the safety and the antitumor effects of DC transduced with a recombinant Ad5 vector and coding for a truncated LMP1 and a full-length LMP2 protein. While the vaccine was well-tolerated, its clinical efficacy was limited; no significant increase in LMP1-specific or LMP2-specific T cells was observed *in vivo*, and only three out of the 16 vaccinated patients showed partial clinical benefits (Chia et al., 2012).

## Optimization of the Anti-EBV Vaccine Strategies

While the first studies on anti-EBV vaccines seem encouraging, improvements with regard to immunogenicity and choice of vaccine epitopes appear necessary, the objective being to envisage a more effective vaccine. New vaccine formulations have consequently been developed.

**FIGURE 8:**
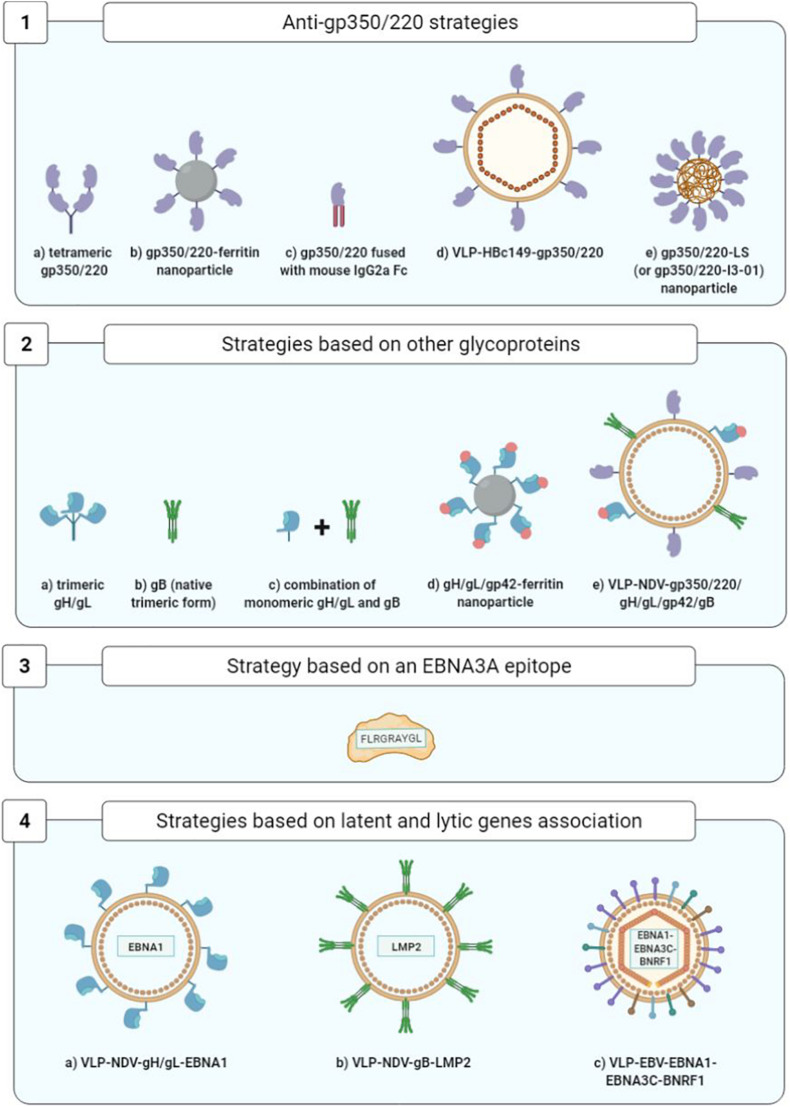
Optimization of prophylactic vaccine strategies. 1a, Cui et al., 2013; 1b, Kanekiyo et al., 2015; 1c, Zhao et al., 2018; 1d, Zhang et al., 2020; 1e, Kang et al., 2021; 2a,2b, Cui et al., 2016; 2c, Cui et al., 2021; 2d, Bu et al., 2019; 2e, Escalante et al., 2020; 3, Elliott et al., 2008; 4a,4b, Perez et al., 2017; 4c, van Zyl et al., 2018.

As concerns prophylactic vaccines, while gp350/220 was the first identified and remains the most widely studied vaccine target, the glycoproteins gH/gL, gp42, and gB have also been identified as neutralizing antibody targets (Sathiyamoorthy et al., 2017; Snijder et al., 2018). In addition, mixed approaches combining EBV lytic cycle and viral cycle proteins are being developed (Figure 8).

**FIGURE 9:**
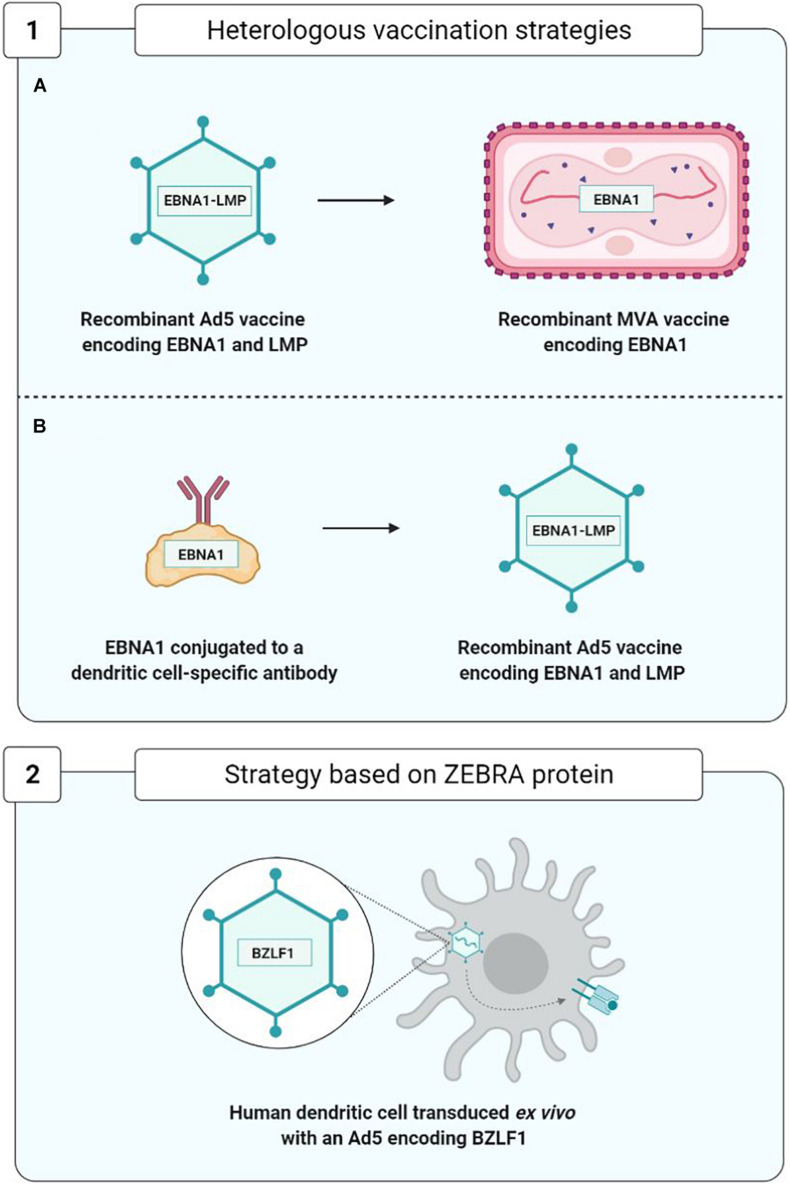
Optimization of therapeutic vaccine strategies. **(1A,1B)**, Rühl et al., 2019; 2, Hartlage et al., 2015.

As concerns therapeutic vaccines, homologous vaccination using the same viral vector for the prime and the boost injections is being superseded by more effective strategies of heterologous vaccination using two distinct vaccine formulations (Figure 9).

### Optimization of the Prophylactic Vaccines

#### Anti-gp350/220 Strategies

Improvement of soluble monomeric gp350 vaccines initially consisted in developing gp350/220 tetramers that succeeded in heightening the levels of anti-gp350/220 neutralizing antibodies in BALB/c mice (Cui et al., 2013).

Another approach consisted in developing self-assembling ferritin nanoparticles expressing an epitope of the gp350/220 binding site at cellular CR2. Using this approach, it became possible to multiply by 10 (in the macaque monkey) and by 100 (in the BALB/c mouse) the anti-gp350/220 antibody titers (Kanekiyo et al., 2015).

Another strategy succeeded in multiplying by 10–100 the titers of neutralizing anti-gp350/220 antibodies in BALB/c mice by fusing the gp350/220 ectodomain to the Fc fragment of mouse IgG2a (Zhao et al., 2018).

Another team developed viral particles without viral DNA termed known as VLPs (virus-like particles); arising from nucleocapsid HBc149 of the hepatitis B virus (VLP-HBc149-gp350/220), they constitute a combination of three epitopes derived from the gp350/220-CR2 binding site. In immunized BALB/c mice, VLPs have yielded high levels of neutralizing antibodies (Zhang et al., 2020).

Lastly, Kang et al. (2021) demonstrated the immunogenicity of the first 425 residues of the gp350/220 ectodomain expressed on two different self-assembled nanoparticles that mimic the shape and size of EBV: lumazine synthase (LS) and I3-01, two capsid-forming enzymes. The gp350/220_1__–__425_-LS and gp350/220_1__–__425_-I3-01 nanoparticles adjuvanted with aluminum hydroxide or MF59 elicited in mice over 133- and 65-fold higher neutralizing antibody titers, respectively, than that induced by the corresponding gp350 monomer (Kang et al., 2021).

While these new strategies have yet to be compared with one another, all of them seem to be more immunogenic than soluble gp350 monomers and represent promising attempts to enhance the immunogenicity of anti-gp350/220 vaccines.

Improvement of anti-gp350/220 vaccines requires not only modifications of gp350/220 structure, but also the use of new adjuvants capable of optimizing anti-gp350/220 immune response. This is particularly the case with glucopyranosyl lipid A-stable emulsion (GLA/SE), which has significantly and durably increased the levels of neutralizing antibodies and anti-gp350/220 T cell response in vaccinated mice and rabbits (Heeke et al., 2016).

#### Strategies Based on the Other EBV Glycoproteins of Interest

The glycoproteins gH/gL and gB assume a preponderant role, not only in EBV entry into B cells, but also (contrarily to gp350/220) into epithelial cells (Snijder et al., 2018).

A study conducted on rabbits immunized with the monomeric or trimeric forms of the gH/gL proteins, the trimeric native form of gB and the monomeric or tetrameric forms of gp350/220 showed that the animals produced higher titers of neutralizing antibodies against the gH/gL and gB glycoproteins than against gp350/220. Moreover, the multimeric forms were more immunogenic than their monomeric counterparts, and whatever its form, the gH/gL glycoprotein was the most immunogenic (Cui et al., 2016).

Five years later, the same team showed that immunization of rabbits with the combination of gH/gL (monomeric form) and gB (trimeric native form) elicited higher neutralizing antibody titers (for both B cells and epithelial cells) than that induced by gH/gL or gB alone. In addition, they demonstrated that sera from rabbits immunized with this combination of gH/gL and gB decreased the EBV load in peripherical blood of humanized mice and protected these mice from death caused by lethal dose EBV challenge (Cui et al., 2021).

Another team compared the humoral responses developed by macaques against ferritin nanoparticles expressing gH/gL glycoproteins alone or combined with gp42, to nanoparticles expressing gp350/220. The gH/gL/gp42-ferritin vaccine complex was found to confer a neutralizing antibody titer highly superior to that of the gp350-ferritin complex. The researchers also noted that while the addition of gp42 to the gH/gL nanoparticles multiplied by four to eight times the titers of the antibodies that neutralize B cell infection, it had no significant impact on the epithelial cells. Finally, association of the gp350-ferritin nanoparticles and gH/gL/gp42-ferritin nanoparticles yielded higher neutralization levels than with gH/gL/gp42-ferritin alone (Bu et al., 2019).

To conclude, a recent study evaluated the injection in rabbits of Newcastle disease virus-like particles (NDV-VLP) expressing the five viral glycoproteins essential to the entry of EBV in target cells (gp350/220, gp42, gH, gL, and gB) in their association with adjuvants (aluminum hydroxide combined with monophosphoryl lipid A). This pentavalent (5-in-1) vaccine stimulated production of antibodies specific to the five glycoproteins capable of neutralizing EBV infection of the B cells as well as the epithelial cells. The IgG anti-gp350/220 and anti-gB levels were markedly higher than the IgG anti-gp42 and anti-gH/gL levels (Escalante et al., 2020).

#### Strategy Based on the EBNA3A Latency Gene

The objective of one original approach toward an anti-EBV prophylactic vaccine has been to prevent IM development stimulating TCD8+ immune cell response directed against EBNA3A and capable of controlling the expansion of EBV-infected B cells. Given the transformative capacity of EBNA3A, an epitope of this viral antigen (FLRGRAYGL) has been used and associated with the tetanic anatoxin as an adjuvant, in an oil-in-water emulsion. The safety and immunogenicity of this vaccine epitope were demonstrated during a phase I clinical trial bringing together 14 healthy adult volunteers, all of whom were EBV-seronegative. They were monitored for up to 12 years after the vaccination, and it was shown that four out of the eight persons having received a low dose of the vaccine (5 μg) were EBV-infected but did not develop IM. As for the two persons vaccinated with a high dose of vaccine epitope (50 μg), one was infected with EBV and developed minimal IM symptoms. Lastly, two out of the four persons having received a placebo were infected with EBV, and one of them developed IM. However, the small study population precludes definitive conclusions on the efficacy of this candidate vaccine in IM prevention (Elliott et al., 2008).

#### Strategies Based on the Association of Latency Genes and the Lytic Cycle at the VLP Surface

In the BALB/c mice, Perez et al. (2017) studied the vaccine combinations gH/gL-EBNA1 and gB-LMP2 expressed by NDV-VLP and compared them to NDV-VLP-gp350/220. The mice immunized with NDV-VLP-gH/gL-EBNA1 or NDV-VLP-gB-LMP2 developed an EBNA1 and LMP2-specific T cell response. The neutralizing antibody titers of these mice were higher than those of the mice immunized with NDV-VLP-gp350/220 (Perez et al., 2017).

Another, even more audacious strategy consisted in utilizing VLPs directly derived from non-infectious and non-oncogenic EBV particles (without viral DNA), modified to express the latency proteins EBNA1 and EBNA3C fused with protein BNRF1 (the main EBV tegument protein) (Pavlova et al., 2013). Injection of these modified EBV-VLPs in humanized mice proved conducive to development of a TCD4+ immune cell response that was specific to both structure proteins (BNRF1) and latency proteins (EBNA1 and EBNA3C) (van Zyl et al., 2018, 2019).

### Optimization of the Therapeutic Vaccines

In order to improve the immunogenicity of anti-EBV therapeutic vaccines based on viral vectors, new approaches to heterologous vaccination have appeared (Figure 9). They consist, during the boost injection, in utilizing a construct different from that of the prime injection. This approach limits the appearance of anti-vector neutralizing antibodies and enhances vaccine efficacy; the two trials carried out by Rühl et al. (2019) constitute a good example.

In the first trial, in the prime injection the team used a recombinant Ad5 viral vector expressing an EBNA1-LMP polyepitope, and in the boost injection, they employed a recombinant and attenuated MVA viral vector expressing the EBNA1 protein.

In another trial, in the prime injection they used a monoclonal antibody protein construct directed against the DEC-205 receptor of the DC attached to protein EBNA1. This construct facilitates the entry and presentation of protein EBNA1 by means of the DCs. The prime injection was boosted by the injection of an Ad5 viral vector expressing the EBNA1-LMP polyepitope.

These two approaches have enabled development of a TCD4+ and TCD8+ EBNA1-specific cell response effectively protecting the mouse from the T and B cell lymphomas expressing EBNA1, thereby justifying their use as therapeutic anti-EBV vaccines in future clinical studies (Rühl et al., 2019).

Another therapeutic vaccination strategy, based on the capacity of the ZEBRA protein to initiate a transition from the viral latency phase to the lytic phase, was used on a hu-PBL-SCID model of mice capable of developing EBV-associated lymphoproliferative diseases (Tang et al., 2016). This vaccine strategy employed human DC transduced with a recombinant Ad5 encoding the ZEBRA protein and enabling the development of ZEBRA-specific TCD8+ cell responses capable of recognizing and eliminating EBV-transformed cells and of significantly delaying the death of mice suffering from lymphoproliferative diseases (Hartlage et al., 2015).

## Discussion

Notwithstanding all the efforts expended, up until now no commercialized anti-EBV vaccine able to prevent infection, IM or cancers associated with EBV has been developed. Moreover, anti-EBV vaccination continues to encounter major obstacles (Balfour, 2014).

As concerns prophylactic vaccines, the objective of sterilizing immunity completely preventing the infection appears difficult to achieve in humans. Indeed, it has been shown than an individual can host multiple EBV strains probably acquired by multiple infections. To put it another way, anti-EBV immunity does not prevent reinfections (Walling et al., 2003). The risk of a vaccine transitorily preventing infection would be to put off primary EBV infection until an age when IM infection is more frequent (Dunmire et al., 2018).

If the objective of an anti-EBV vaccine is to limit occurrence of the cancers associated with the virus, the low incidence of these cancers and the sizable time they take to appear considerably complicates clinical trials capable of proving vaccinal efficacy.

Last but not least, another major obstacle consists in the absence of efficient animal models, affecting not only the development of prophylactic vaccines, but also research on therapeutic vaccines (Rühl et al., 2020).

Present-day hopes for an anti-EBV vaccine reside in a combination of several strategies and on the use of several latency and lytic cycle proteins that could induce a broad spectrum of neutralizing antibodies and TCD8+ and TCD4+ cellular response. The objective being to block the essential steps of the viral cycle, from the entry through the persistence of the target cells, and to provide protection against EBV infection (prophylactic effect) and the associated diseases (therapeutic effect).

## Author Contributions

VJ-P and RG drafted the manuscript. All authors contributed to manuscript revision, read, and approved the submitted version.

## Conflict of Interest

The authors declare that the research was conducted in the absence of any commercial or financial relationships that could be construed as a potential conflict of interest.

## Abbreviations

Ad5: type 5 adenovirus
CR2: complement receptor type 2
CTLD: C-type lectin domain
DC: dendritic cell
EBER: Epstein-Barr encoded small RNA
EBNA: Epstein-Barr nuclear antigen
EBV: Epstein-Barr virus
EphA2: ephrin receptor tyrosine kinase A2
FBN: fibronectin region
g, gp: glycoprotein
GLA/SE: glucopyranosyl lipid A-stable emulsion
HLA: human leukocyte antigen
IM: infectious mononucleosis
LBD: ligand binding domain
LMP: latent membrane protein
miRNA: microRNAs
MVA: modified vaccinia virus Ankara
NPC: nasopharyngeal carcinoma
NRP1: neuropilin 1
PTLD: post-transplant lymphoproliferative disease
VCA: viral capsid antigen
VLP: virus-like particle
VV: vaccinia virus.
